# Diagnostic value of applying preoperative breast ultrasound and clinicopathologic features to predict axillary lymph node burden in early invasive breast cancer: a study of 1247 patients

**DOI:** 10.1186/s12885-024-11853-2

**Published:** 2024-01-22

**Authors:** Hua Shao, Yixin Sun, Ziyue Na, Hui Jing, Bo Li, Qiucheng Wang, Cui Zhang, Wen Cheng

**Affiliations:** 1 Department of Medical Ultrasound, Harbin Medical University Cancer Hospital, 150081 Harbin, Heilongjiang China; 2 Department of Interventional Ultrasound, Harbin Medical University Cancer Hospital, 150081 Harbin, Heilongjiang China

**Keywords:** Ultrasound, Clinicopathologic features, Lymph node burden, Early invasive breast cancer

## Abstract

**Background:**

Since the Z0011 trial, the assessment of axillary lymph node status has been redirected from the previous assessment of the occurrence of lymph node metastasis alone to the assessment of the degree of lymph node loading. Our aim was to apply preoperative breast ultrasound and clinicopathological features to predict the diagnostic value of axillary lymph node load in early invasive breast cancer.

**Methods:**

The 1247 lesions were divided into a high lymph node burden group and a limited lymph node burden group according to axillary lymph node status. Univariate and multifactorial analyses were used to predict the differences in clinicopathological characteristics and breast ultrasound characteristics between the two groups with high and limited lymph node burden. Pathological findings were used as the gold standard.

**Results:**

Univariate analysis showed significant differences in ki-67, maximum diameter (MD), lesion distance from the nipple, lesion distance from the skin, MS, and some characteristic ultrasound features (*P* < 0.05). In multifactorial analysis, the ultrasound features of breast tumors that were associated with a high lymph node burden at the axilla included MD (odds ratio [OR], 1.043; *P* < 0.001), shape (OR, 2.422; *P* = 0.0018), hyperechoic halo (OR, 2.546; *P* < 0.001), shadowing in posterior features (OR, 2.155; *P* = 0.007), and suspicious lymph nodes on axillary ultrasound (OR, 1.418; *P* = 0.031). The five risk factors were used to build the predictive model, and it achieved an area under the receiver operating characteristic (ROC) curve (AUC) of 0.702.

**Conclusion:**

Breast ultrasound features and clinicopathological features are better predictors of high lymph node burden in early invasive breast cancer, and this prediction helps to develop more effective treatment plans.

## Background

The preoperative status of axillary lymph node (ALN) in early-stage breast cancer is very important and will affect treatment options and prognosis [1]. Breast cancer surgical treatment philosophy is shifting from “maximum tolerable” to “minimum effective treatment [2]. Sentinel lymph node biopsy (SLNB) has replaced axillary lymph node dissection (ALND) as the standard procedure for lymph node staging in breast cancer with clinically negative axillary lymph nodes. If metastatic axillary lymph nodes are observed by SLNB, axillary lymph node dissection is often required, and ALND does not improve patient survival or reduce the rate of local recurrence [3, 4]. However, it can increase complications such as lymphedema, upper extremity sensory abnormalities, and limitation of movement, which can reduce the quality of patient survival [5]. Based on the results of the American College of Surgeons in Oncology (ACOSOG) Z0011 study, the National Comprehensive Cancer Network (NCCN) guidelines recommend that patients with stage T1-2 breast cancer with only one or two positive sentinel lymph nodes who underwent breast-conserving surgery and postoperative whole-breast radiotherapy should be exempted from axillary lymph node dissection [6]. Since then, the assessment of axillary lymph node status has been redirected from simply assessing the presence of lymph node metastases to assessing the degree of lymph node tumor burden. The goal of axillary imaging is to predict high lymph node burden (≥ 3 metastatic ALNs) rather than to predict lymph node metastasis. Low lymph node burden is often defined as one to two metastatic lymph nodes, while three or more metastatic lymph nodes are considered high lymph node burden [7]. Therefore, preoperative differentiation between patients with low nodal burden (LNB) and patients with high nodal burden (HNB) can help guide individualized axillary lymph node surgery.

Ultrasound is widely used as a non-invasive and convenient tool for preoperative assessment of the primary lesion of breast cancer and the status of axillary lymph nodes. Conventional axillary ultrasound can predict ALN status based on changes in the cortical morphological features of ALN [8]. Different diagnostic criteria may lead to unnecessary biopsies in patients with negative lymph nodes or false-negative results in malignant ALNS [9]. In addition, early axillary lymph node metastasis (ALNM) often does not cause structural or size changes on ultrasound [10]. It is widely accepted that the occurrence of axillary lymph node metastasis depends primarily on the biological behavior of the primary breast tumor. Recent studies on nomograms predicting axillary lymph node metastasis have re-evaluated the role of clinicopathological features of the primary tumor in predicting axillary lymph node metastasis [11, 12]. However, the relationship between clinicopathological features and breast ultrasound characteristics and axillary lymph node load is unclear. The purpose of this study was to evaluate the value of clinicopathological features combined with breast and axillary lymph node ultrasound features in predicting axillary lymph node burden.

## Methods

### Ethical statement

This retrospective study was approved by the Institutional Review Board of Hospital. Informed consent was not required because of the retrospective nature of the cohort study.

### Patients

**Fig. 1:**
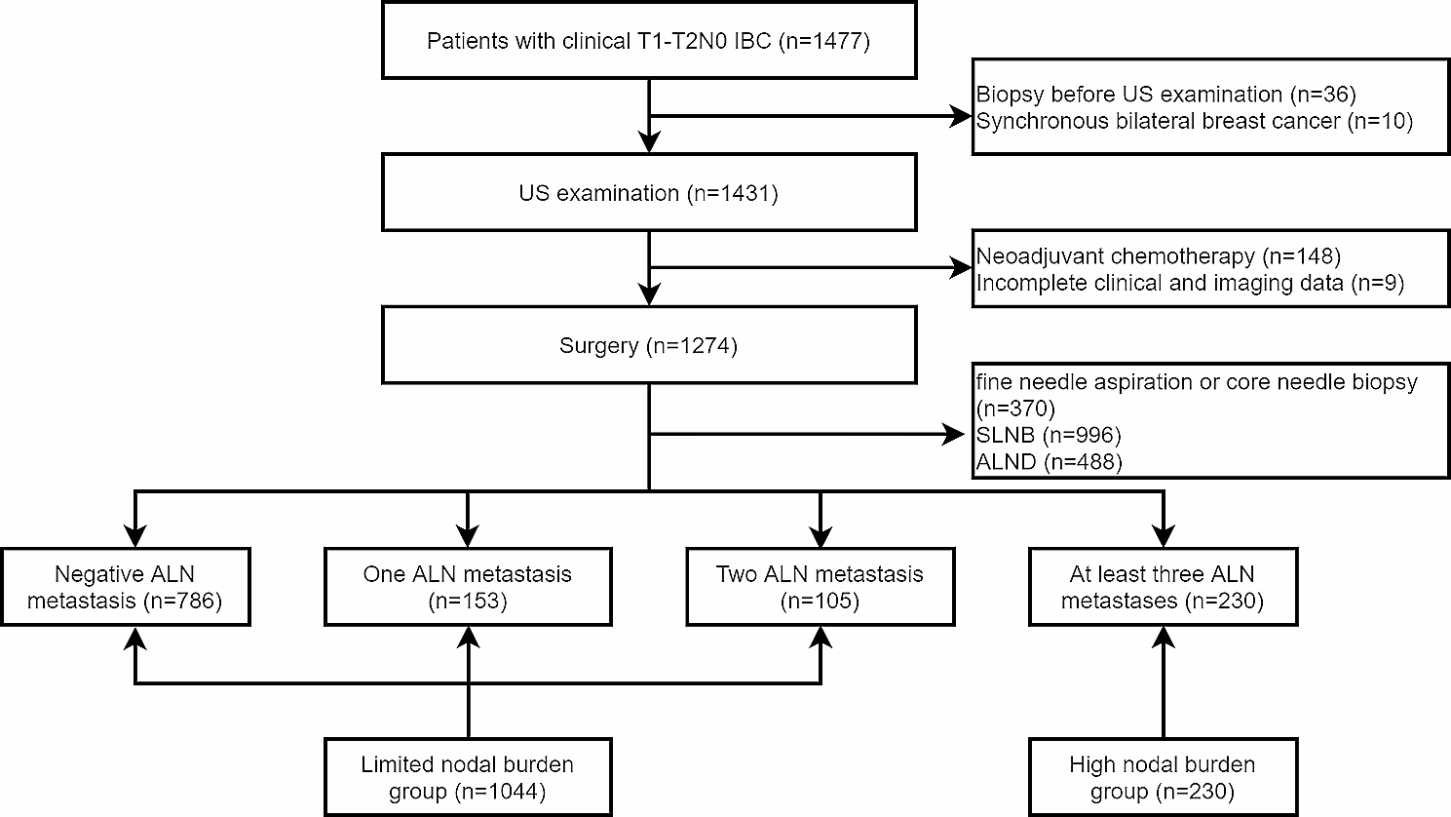
Flowchart of procedures in the breast lesion selection. IBC = invasive breast cancer, US = ultrasound, ALN = axillary lymph node

This study includes patients with clinical T1-T2N0 invasive breast cancer (IBC) diagnosed by surgery or biopsy specimens between January 2018 and December 2022. All patients underwent ultrasound examinations before surgery or biopsy. Nowikiewicz T et al [13] concluded that the shorter the time between ultrasound examination and pre-surgery the more accurate the assessment of the extent of metastasis in lymph nodes. Therefore, Breast ultrasound within two weeks before surgery. The patient’s series of ultrasound examinations are performed by a skilled radiologist who records the patient’s ultrasound image. The patient collection process is shown in Fig. 1. For patients with abnormal looking axillary lymph nodes on ultrasound, a biopsy (fine needle aspiration or core needle biopsy) of the most suspicious lymph node would be offered. ALND was performed if the lymph node had metastasis, and SLNB was performed intraoperatively if there was no metastasis. Patients with no suspicious lymph nodes detected by ultrasound. Then SLNB was performed intraoperatively. ALND was performed if SLNB detected metastasis in axillary lymph nodes. Mastectomy and breast-conserving surgery specimens were tested for estrogen receptor (ER), progesterone receptor (PR), human epidermal growth factor receptor 2 (HER-2), Ki-67 and P53. Patient inclusion criteria are as follows: [1] Pathologically confirmed breast cancer with only one lesion, [2] T1-T2 stage breast cancer without distant metastasis, [3] no neoadjuvant chemotherapy or radiotherapy prior to US examination, [4] ALN status clearly confirmed by SLNB or ALND, and [5] complete data and clinical information.

### Ultrasound examination

Ultrasound is performed with high-resolution ultrasound equipment, including the Philips.

EPIQ7 (Philips Ultrasound, Bothell, Washington), Aplio i900 (Canon Medical Systems USA, Inc., Tustin, CA), Acuson S3000 (Siemens Healthcare, Erlangen, Germany), and the Resona 7 (Mindray, Shenzhen, China). All machines are equipped with high frequency (5–14 MHz) linear array transducers. Preoperative ultrasound was performed by one of five breast imaging specialists with more than 8 years of clinical experience. During breast ultrasound, the maximum diameter of the breast cancer lesion was measured to determine preoperative clinical T-stage. Ultrasound features were reviewed retrospectively by two experts independently. In case of disagreement, two other experts joined in to reach consensus and resolved the discrepancy.

The ultrasonographic features of primary breast lesions were analyzed using the 5th American College of Radiology Breast Imaging Reporting and Data System (BI-RADS) lexicon. Documented imaging features include MD, location (lower inner quadrant, upper outer quadrant, lower outer quadrant, upper inner quadrant), shape (regular, irregular), margins (circumscribe, Indistinct, angular, microlobulated, spiculated), orientation (parallel, nonparallel), posterior features (no features, posterior acoustic enhancement, shadowing, combined pattern), calcifications (absent, present), hyperechoic halo (negative, positive). After breast ultrasound, axillary lymph node scans are routinely performed by the same radiologist, and preoperative findings are classified as normal or suspicious. Axillary ultrasound findings were considered normal when no suspicious lymph nodes were seen in the axilla, and suspicious when there were abnormal lymph nodes in the axilla with at least one suspicious sign. Lymph nodes with the following findings were defined as suspicious [14]: hypoechoic lymph nodes with a long-to-short (L/S) ratio less than 2.0, cortical thickness greater than or equal to 3 mm with eccentric thickening, and complete or partial effacement of the fat hilum.

### Histopathological analysis

The ER, PR, Ki-67 and p53 status were determined by immunohistochemistry, and HER-2 was determined by immunohistochemistry or fluorescence in situ hybridization (FISH). P53, ER, PR and HER-2 status were defined as follows: Ki-67 status (negative < 14%,positive ≥ 14%),P53 status (negative < 10%,positive ≥ 10%),ER, PR status ( negative ≤ 1%,positive1>%),HER-2 status (negative 0 or 1+,positive 3+,borderline 2+). When the HER-2 status was 2+, FISH was performed for the final determination. Patients were classified into four molecular subtypes (MS) based on previously validated clinicopathological criteria [15]. Molecular subtype (MS), that is, luminal A, luminal B, HER-2 overexpression, triple negative subtype (TN). Axillary lymph node status was recorded.

### Data analysis

Retrieval of clinical information from the electronic medical record (age, marital status, fertility status, menopausal status and other clinical factors). Evaluation of pathological grading, pathological type, ER, PR, HER-2 and Ki-67 expression, molecular subtypes and lymph node metastasis of breast cancer based on histopathology reports. All lesions were divided into a limited lymph node burden group (< 3 metastatic ALN) and a high lymph node burden group (≥ 3 metastatic ALN) based on pathological findings. Univariate analysis was performed to compare US characteristics and various clinicopathological factors between the two groups. The correlation of each variable with high lymph node burden was investigated using univariate and multifactorial analyses.

### Statistical analysis

SPSS 20.0 software was used for statistical analysis. Means ± standard deviations were used to describe measurement data that conformed to a normal distribution. The t-test was used for comparison between groups. If the data did not conform to a normal distribution, the median and quartiles (Q1, Q3) were used for statistical description. Comparisons between groups were made using the rank sum test. Count data were described as counts and percentages, and the chi-square test was used for comparison between groups. Univariate logistic regression analysis was performed, and covariates with *P* < 0.05 were considered significant (to avoid eliminating significant variables). Variables found to be significant in the univariate analysis were included in the multivariate analysis.

## Results

**Fig. 2:**
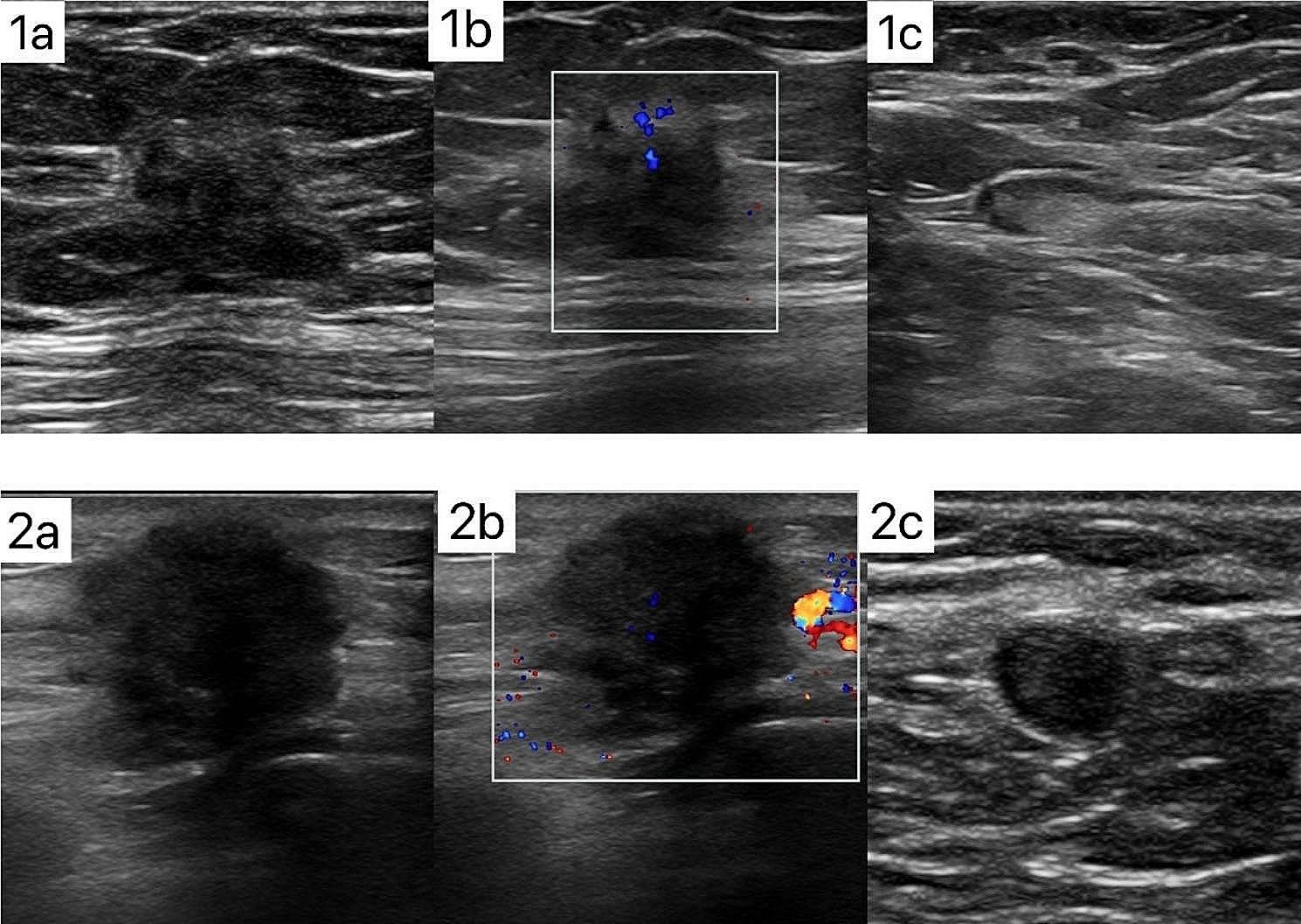
**1a**. US image of 31-year-old woman in stage I (MD = 1.2 cm) breast cancer with limited lymph node burden. US detected a hypoechoic lesion on outer quadrant in the right breast with irregular shape, indistinct margin, posterior no feature and hyperechoic halo. **1b**. CDFI: A small amount of punctate blood flow signal is visible. **1c**. Axillary ultrasound shows normal lymph nodes. **2a**. US image of 43-year-old woman in stage II (MD = 2.8 cm) breast cancer with high lymph node burden. US detected a hypoechoic lesion on outer quadrant in the left breast with irregular shape, spiculated margin, posterior feature shadowing and hyperechoic halo. **2b**. CDFI: penetrating branch blood flow signal is visible. **2c**. Axillary ultrasound shows suspicious lymph nodes

In this study, there were a total of 1274 female patients with 1274 early-stage breast cancer (EBC) lesions. Histological type is invasive ductal carcinoma. The mean age of the patients was 55.0 ± 10.29 years (range 24–86 years), and the mean lesion MD measured by US was 2.405 ± 1.0313 cm (range 0.5-5.0 cm). Limited lymph node burden 1044 patients (81.95%,1044/1274), of which 786 patients had negative metastases, 153 patients had 1 metastasis, 105 patients had 2 metastases, and 230 patients had high lymph node burden (18.05%,230/1274). There were 544 patients (42.70%,544/1274) in stage I and 730 patients (57.30%,730/1274) in stage II. A comparison of clinicopathological factors and US characteristics of IBC lesions with and without high lymph node burden is shown in Tables 1 and 2. Ki-67, MS, MD, morphology, hyperechoic halo, posterior features, and suspicious lymph nodes on axillary ultrasonography were significantly different between the high and limited nodal burden group (*P* < 0.05). The lesions in the high nodal group were significantly larger than those in the limited nodal burden group 2.793 ± 1.112 cm than 2.319 ± 0.995 cm (*P* < 0.001). Lesions with shorter distances to the skin [0.425 ± 0.277 vs. 0.478 ± 0.258] and shorter distances to the nipple[2.35 ± 2.042 cm vs. 2.65 ± 2.117 cm] were more prone to occur in the high nodal burden group (*P* < 0.05). There was little difference between the two classifications with respect to age, marital status, Fertility status, Menopause, ER, PR, HER2, p53, and location, among others. The typical patients are demonstrated in Fig. 2.

**Table 1:** Disparities in clinicopathological features amongst patients with the high and limited nodal burden

Variables	Total (*n* = 1274)	Limited nodal burden(%) (*n* = 1044)	High nodal burden (%) (*n* = 230)	χ2	P值
Age				0.104	0.748
≤ 50	466	384(36.8)	82(35.7)		
>50	808	660(63.2)	148(64.3)		
Marital status				0.001	0.977
Unmarried	8	5(0.5)	3(1.3)		
Married	1266	1039(99.5)	227(98.7)		
Fertility status				0.084	0.773
No	27	16(1.5)	11(4.8)		
Yes	1247	1028(98.5)	219(95.2)		
Menopause				2.138	0.144
No	522	449(43.0)	73(31.7)		
Yes	752	595(57.0)	157(68.3)		
Histologic grade				1.233	0.267
I级	149	127(12.2)	22(9.6)		
II-III级	1125	917(87.8)	208(90.4)		
Ki-67				7.500	0.006
Negative	425	366(35.1)	59(25.7)		
Positive	849	678(64.9)	171(74.3)		
P53				0.303	0.582
Negative	407	330(31.6)	77(33.5)		
Positive	867	714(68.4)	153(66.5)		
ER				0.123	0.726
Negative	333	275(26.3)	58(25.2)		
Positive	941	769(73.7)	172(74.8)		
PR				0.044	0.833
Negative	419	342(32.8)	77(33.5)		
Positive	855	702(67.2)	153(66.5)		
HER-2				1.073	0.300
Negative	873	722(69.2)	151(65.7)		
Positive	401	322(30.8)	79(34.3)		
MS				11.044	0.011
Luminal A	365	317(30.4)	48(20.9)		
Luminal B	591	464(44.4)	127(55.2)		
Her-2 overexpression	140	114(10.9)	26(11.3)		
TN	178	149(14.3)	29(12.6)		
Location				11.008	0.012
lower inner quadrant	71	60(5.8)	11(4.8)		
upper outer quadrant	685	545(52.2)	140(60.9		
lower outer quadrant	198	158(15.1)	40(17.3)		
upper inner quadrant	320	281(26.9)	39(17.0)		

EBC Early-stage breast cancer, ER Estrogen-receptor, PR Progesterone-receptor, HER-2 Human epidermal growth factor receptor 2, MS Molecular subtype, TN Triple negative

**Table 2:** Univariate analysis of the variations in ultrasonic characteristics between the limited nodal burden group and the high nodal burden group

US features	Total (*n* = 1274)	Limited nodal burden(%) (*n* = 1044)	High nodal burden (%) (*n* = 230)	P值
Distance to nipple	2.60 ± 2.106	2.65 ± 2.117	2.35 ± 2.042	0.045
Distance to skin	0.468 ± 0.262	0.478 ± 0.258	0.425 ± 0.277	0.005
MD	2.404 ± 1.033	2.319 ± 0.995	2.793 ± 1.112	< 0.001
Orientation				0.483
Parallel	1159	947(90.7)	212(92.2)	
Nonparallel	115	97(9.3)	18(7.8)	
Margin				0.087
Circumscribe	105	95(9.1)	10(4.3)	
Indistinct	951	770(73.8)	181(78.7)	
Angular	26	22(2.1)	4(1.7)	
Microlobulated	13	9(0.9)	4(1.7	
Spiculated	179	148(14.1)	31(13.6)	
Hyperechoic halo				< 0.001
Negative	942	804(77.0)	138(60.0)	
Positive	332	240(23.0)	92(40.0)	
Posterior features				< 0.001
No features	1043	845(80.9)	198(86.1)	
Enhancement	108	104(10.0)	4(1.7)	
Shadowing	52	45(4.3)	23(10.0)	
Combined pattern	71	50(4.8)	5(2.2)	
Calcifications				0.261
Absent	524	437(41.9)	87(37.8)	
Present	750	607(58.1)	143(62.2)	
Shape				0.002
Regular	119	110(10.5)	9(3.9)	
Irregular	1155	934(89.5)	221(96.1)	
Axillary US				0.001
Normal	904	761(72.9)	143(62.2)	
Suspicious	370	283(27.1)	87(37.8)	

EBC Early-stage breast cancer, MD Maximum diameter, US ultrasound

Among 1274 patients, preoperative axillary ultrasound findings showed normal lymph nodes in 904 (71.0%) and suspicious lymph nodes in 370 (29.0%) (Table 2). The incidence of high lymph node burden was higher in patients with suspicious axillary ultrasound findings than in those with negative axillary ultrasound findings (23.5% and 15.8%, respectively; *P* = 0.001). Of the 904 patients with normal axillary ultrasound findings, limited lymph node load was found in 761 (84.2%) and high lymph node load in 143 (15.8%) in the final pathology (Table 2). When suspicious lymph nodes were detected on axillary ultrasound, limited lymph node load remained in 76.5% (283/370) of patients. The rate of false positive axillary ultrasound showing limited lymph node burden was 27.1% (283/1044).

**Fig. 3:**
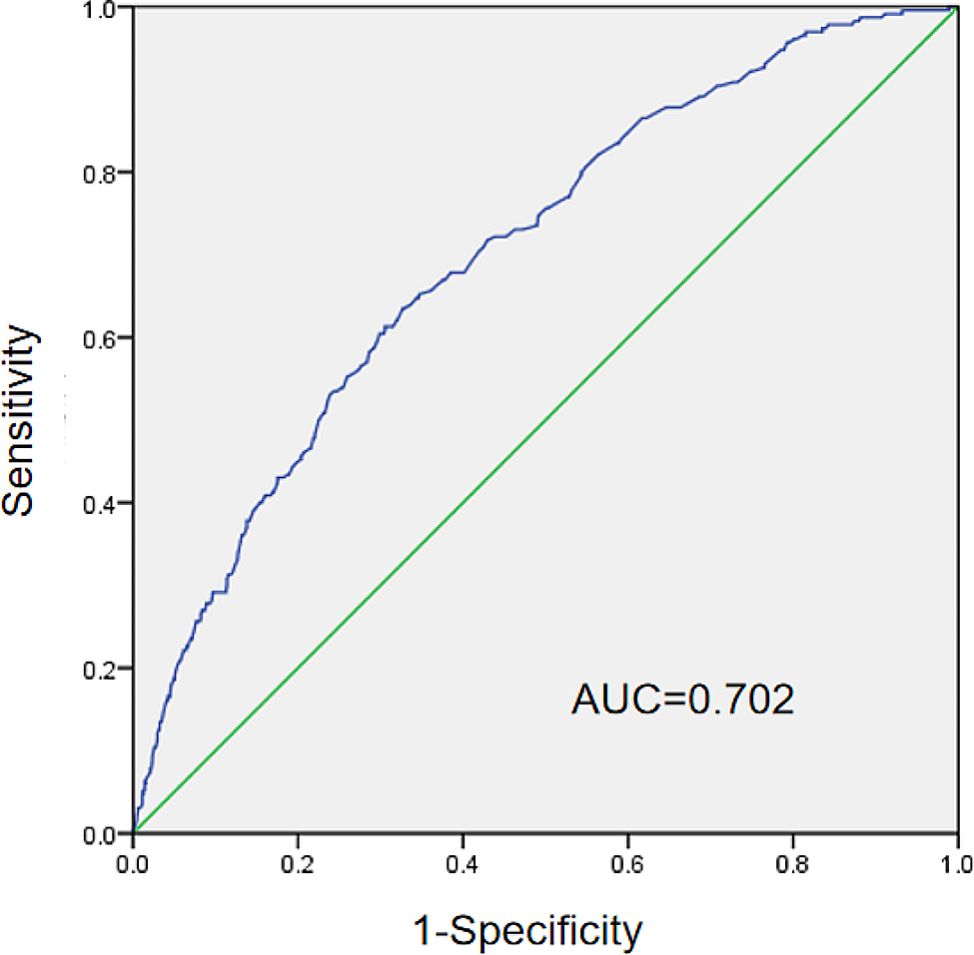
Receiver operating characteristic curve for MD, shape, hyperechoic halo, posterior features and suspicious axillary US performance on high lymph node burden. AUC indicates area under the curve

Univariate logistic regression analysis revealed Ki-67 positivity, MD and US characteristics (Table 3) as independent predictors of IBC with the high nodal burden group (*P* < 0.05). Luminal B was a protective factor (*P* = 0.001) against the high nodal burden group relative to the luminal A subtype. Posterior acoustic enhancement features of the primary tumor was protective factor for the high nodal burden relative to the not features (*P* < 0.001) (Table 3). The initial input for the multiple logistic regression analysis was based on significant variables found in the univariate analysis (*P* < 0.05). Multifactorial analysis revealed tumor size (*P* < 0.001), hyperechoic halo (*P* < 0.001), shape (*P* = 0.018), posterior acoustic enhancement (*P* = 0.002), shadowing (*P* = 0.007), and suspicious axillary ultrasound performance (*P* = 0.031) as independent predictors associated with high lymph node burden; ki67, molecular subtype, and combined pattern in posterior features were not significant factors (Table 3). The multivariate regression model was built as follows: Y = − 1.513 + 0.038 × MD + 0.882 × shape + 0.678 × hyperechoic halo + 0.347× axillary US + (0.733 × enhancement or + 0.748 ×shadowing or– 1.615 × combined pattern). A Receiver operating characteristic curve was drawn, and the area under the curve was 0.702 (Fig. 3).

**Table 3:** Univariate and multivariate analysis of the risk factors in EBC* with the high nodal burden group

Variables	Univariate analysis			Multivariate analysis	
	P	OR(95%CI)		P	OR(95%CI)
MD	< 0.001	0.076(1.029,1.057)		< 0.001	1.043(1.028,1.058)
Ki-67	0.006	1.565(1.134,2.159)		0.760	1.121(0.539,2.333)
MS					
Luminal A	Ref	Ref			
Luminal B	0.001	1.808(1.259,2.595)		0.796	1.133(0.495,2.503)
Her-2 overexpression	0.125	1.506(0.893,2.541)		0.073	0.431(0.172,1.081)
TN	0.325	1.285(0.779,2.120)		0.787	1.121(0.491,2.555)
Hyperechoic halo	< 0.001	2.233(1.653,3.017)		< 0.001	2.546(1.774,3.655)
Posterior features					
No features	Ref	Ref			
Enhancement	< 0.001	0.164(0.060,0.451)		0.002	0.193(0.069,0.542)
Shadowing	0.004	2.181(1.289,3.690)		0.007	2.155(1.234,3.762)
Combined pattern	0.073	0.427(0.168,1.084)		0.123	0.464(0.175,1.233)
Shape	0.003	2.892(1.443,5.796)		0.018	2.422 (1.167,5.025)
Axillary US	< 0.001	0.188(1.213,2.207)		0.031	1.418(1.032,1.949)

EBC Early-stage breast cancer, ER Estrogen-receptor, PR Progesterone-receptor, HER-2 Human epidermal growth factor receptor 2, MS Molecular subtype, TN Triple negative,US ultrasound

## Discussion

Since the ACOSOG-Z0011 trial, the identification of high axillary lymph node burden (≥ 3 tumor-involved lymph nodes) is crucial for systemic treatment of breast cancer. Ultrasound examination has the characteristics of high sensitivity and high predictive value of positive results [16]. However, ultrasound examination results are often influenced by technical limitations and subjective factors. Direct observation of axillary lymph node metastasis using ultrasound can lead to false negative or false positive results. In this study, ultrasound examination was used to directly observe the primary lesion and predict lymph node burden. In this study, using high axillary lymph node burden as a response variable, we found that high axillary lymph node burden in early invasive breast cancer was associated with multiple clinicopathological variables. This included Ki-67 positivity, MD, molecular subtype, distance of the mass from the nipple, distance of the mass from the skin, tumor hyperechoic halo, posterior echogenic features, shape of the primary tumor, and suspicious axillary lymph nodes on ultrasonography. Multifactorial logistic regression analysis showed that tumor size, hyperechoic halo around the mass, shadowing in posterior features, irregular shape of the primary tumor, and suspicious lymph nodes on ultrasonography were independent risk factors for high lymph node burden.

Lesion size in this study was measured by preoperative ultrasound rather than postoperative pathology, which was done to enable preoperative assessment of lymph node burden. In some studies, tumor size was a risk factor for axillary lymph node metastasis [17, 18]. As confirmed by our results, the incidence of high axillary lymph node burden was relatively higher in cancer patients with larger tumor size (OR, 1.043; 95% CI, 1.028,1.058). Breast cancer cells can migrate to the ALN through the lymphatic plexus and lymphatic network within the breast parenchyma and interstitium. Inconsistent tumor margins may promote infiltration of tumor cells into adjacent tissues at different growth rates. This may result in larger tumors being more associated with high lymph node load [19, 20].

The mammary gland has an embryologic origin in the ectoderm and eventually develops entirely within the superficial fascia of the skin. The areolar lymphatic plexus on the outer surface of the breast anastomoses with the superficial cutaneous lymphatic network overlying the skin, and the parenchymal lymphatics accompany the milk ducts and empty centripetally into the dense subareolar plexus, where lymph from all parts of the breast converges, then forms a pooled lymphatic trunk that leaves the areolar area and travels toward the surface of the axillary lymph nodes [21–24]. In the study by Jia-wei Li et al [7], the presence of lymphovascular invasion was 23.52 times more likely to be associated with high lymph node tumor burden, and the presence of papillary invasion was 2.93 times more likely to be associated with high lymph node burden. This would explain in our study why high lymph node burden is more likely to occur with primary tumour closer to skin and nipple.

Ki-67 protein expression has been shown to be associated with cell proliferation and cell cycle activity phase. In general, high levels of Ki-67 expression are strongly associated with high proliferation and poor prognosis and are important predictors of ALNM [25, 26]. Ki-67 positivity in the high lymph node burden group, 171 patients (20.1%), accounted for a high percentage (*P* = 0.006). Ki-67 positivity was a significant predictor of high lymph node burden in univariate analysis.

The main reasons for the irregularity, borderless edges and shadowing of the primary lesion of breast cancer are the rapid proliferation of cancerous tissue, the high content of collagen fibers in the interstitium and the infiltration of adjacent tissues [27]. Many previous studies have shown that posterior characteristic shadowing often suggests the possibility of malignant lesions [28, 29]. Posterior shadowing is caused by increased and disturbed arrangement of collagen fibers in the tumor interstitium, and lesions with posterior shadowing imply slow growth and lower tissue grade [30]. The tumor growth cycle is longer and often not easily detected, leaving more time for axillary metastasis. The histopathological features of hyperechoic halo are: cancer cells infiltrate adipose tissue, mixed adipose tissue, cancer cells and fibroblastic interstitium, which is caused by direct infiltration of cancer tissue. To some extent, it reflects the degree of cancer cell invasion and is an important indicator of poor prognosis. Halo or borderline echogenicity is recognized as an important indicator of malignancy [31, 32]. It is an ill-defined echogenic band located on the surface of the lesion, representing the burr margin of the tumor and the invasive margin of cancer cells, lymphocytes, histiocytes and fibrous connective tissue surrounding the infiltrating malignant tumor [31–33]. It was reported that the wider the hyperechoic halo, the worse the prognosis. The above findings argue for our findings from a pathological point of view. In our study, lesions with characteristic posterior shadowing and hyperechoic halo were more likely to have high lymph node burden than lesions without these features.

The results of this study showed a statistically significant difference between the molecular subtypes, limited lymph node burden and high lymph node burden (*P* = 0.011). luminall A is the more common molecular subtype of breast cancer, which is very sensitive to endocrine therapy and has a better prognosis compared to other subtypes [34]. Luminall B tumor is rich in blood vessels and has a high risk of metastasis. The cancer cells differentiate rapidly and infiltrate the surrounding tissues, and are very likely to infiltrate the axillary lymph nodes, resulting in high lymph node burden. In late stage, the invaded lymph nodes may change in shape and texture and become hypoechoic, and multiple lymph nodes may become calcified and necrotic after fusion [34–36]. Therefore, the malignancy of Luminall B breast cancer is higher than that of Luminall A. The risk of developing high lymph node load was 1.808 times higher in Luminall B compared to Luminall A.

In axillary ultrasonography, morphological changes in the cortex and lymph nodes with hilum absence are considered suspicious. Since metastatic cells live in the cortex of lymph nodes [37, 38], morphologic changes in the cortex are known to be a marker of metastasis. Previous studies also reported that patients with suspicious lymph nodes identified by axillary ultrasound were more likely to have three or more metastatic axillary lymph nodes on final pathology compared with those who were negative [39, 40]. Our findings are consistent with these findings. Patients with suspicious axillary lymph nodes identified on ultrasound were more likely to have a high lymph node burden than patients with normal axillary lymph nodes (OR, 1.418;95%CI, 1.032–1.949)。.

This study enrolled a large sample of early invasive ductal carcinoma with more definitive data, but there are some limitations. First, this is a single-center retrospective study that included only patients with negative SLNB or patients who underwent ALND post positive SLNB; SLNB-negative patients did not undergo further ALND, and there is a possibility of false-negative SLNB; however, this inherent limitation is unavoidable because omitting ALND in SLNB-negative patients is considered safe [41]. There may be selection bias, and it is necessary to expand the sample size for prospective multicenter studies in the future. Second, the measurement of tumor and lymph node size and morphological characteristics by ultrasound technology is subjective, and there may be some measurement errors by different operators or different machines, so more objective and quantitative indicators are still needed. Third, the assessment of the US features of breast tumors was based on a retrospective review of stored still images, which may have caused missed or misinterpreted information.

Our prediction model showed moderate predictive efficacy with an AUC of 0.702. This result is similar to recent studies that investigated the potential value of US characteristics of breast lesions in predicting high lymph node burden, reporting AUCs ranging from 0.678 to 0.876 [42–44]. There are a number of studies that have used US features of breast cancer and ALN to evaluate ALNM and have shown that tumor features correlate with lymph node metastasis [10, 45]. However, there are relatively few studies using tumor characteristics with high lymph node burden. These clinicopathologic features should be considered along with the ultrasound features of the primary lesion when assessing axillary lymph node tumor load and provide additional information for adjuvant therapy. In particular, the risk of high lymph node burden is relatively low in patients with clinical stage T1-2 cancer and negative axillary ultrasound. Preoperative ultrasound characterization of lesions and pathologic findings help identify patients at minimal risk for high lymph node burden and add to the discussion of ALND. In this study, patients who were able to undergo breast-conserving surgery benefited significantly. Primary breast cancer lesions should be examined preoperatively with ultrasound and axillary lymph node status can be initially determined. Preoperatively, this provides additional useful information for patients with limited lymph node burden.

In conclusion, preoperative ultrasonography is indispensable in breast cancer screening due to its ease of operation, real-time dynamics, and easy accessibility. Axillary lymph node loading status is predicted by observing the ultrasound presentation of breast tumors and clinicopathological factors. Although the results based on US examination cannot fundamentally change the decision of SLNB and the surgical approach of EBC. However, it can provide more clinical reference.

## Data Availability

The datasets used and/or analyzed during the current study are available from the corresponding author on reasonable request.

## Abbreviations

MD: Maximum diameter
OR: Odds ratio
ROC: Receiver operating characteristic
AUC: Area under the receiver operating characteristic (ROC) curve
ALN: Axillary lymph node
SLNB: Sentinel lymph node biopsy
ALND: Axillary lymph node dissection
LNB: Low nodal burden
HNB: High nodal burden
ALNM: Axillary lymph node metastasis
IBC: Invasive breast cancer
ER: Estrogen receptor
PR: Progesterone receptor
HER-2: Human epidermal growth factor receptor 2
US: Ultrasound
TN: Triple negative
EBC: Early-stage breast cancer
